# Exploring DNA methylation within the CYP17A gene as a potential mediator between childhood adversity and stress-related phenotypes in schizophrenia

**DOI:** 10.1192/j.eurpsy.2022.517

**Published:** 2022-09-01

**Authors:** M. Alfimova, N. Kondratyev, A. Golov, M. Gabaeva, V. Plakunova, V. Golimbet

**Affiliations:** Mental Health Research Center, Clinical Genetics Laboratory, Moscow, Russian Federation

**Keywords:** schizophrénia, DNA methylation, CYP17A gene, childhood adversity

## Abstract

**Introduction:**

Stress caused by childhood adversity (CA) is known to contribute to schizophrenia risk and symptoms. Its effects might be mediated by epigenetic mechanisms, specifically DNA methylation (meDNA) within relevant genes, and predominantly influence the hippocampus and prefrontal cortex (PFC). *CYP17A1* is a candidate, as it situates within a schizophrenia risk locus and is involved in glucocorticoid synthesis.

**Objectives:**

To explore meDNA within *CYP17A* and its relations to hippocampus- and PFC-dependent schizophrenia symptoms: depression and deficits of declarative memory and executive functions.

**Methods:**

We assessed meDNA at each CpG within a *CYP17A* fragment (chr10:104594471-104595887, hg19) in blood of 66 schizophrenia patients using the third-generation sequencing. Immediate memory, depression, cognitive shifting and cognitive inhibition (CI) were assessed with the RAVLT, PANSS, TMT-B and Stroop word-color test, respectively. ANCOVA and regression models adjusted for sex and age were applied to explore the relations between the phenotypes, local haplotype, meDNA and CA, defined as the presence of parental alcoholism or psychiatric illness.

**Results:**

MeDNA at CpG-SNP rs3781286 correlated with CI (corrected p=0.01). However, there were no main or interaction effects of CA either on meDNA at this site or on CI. Both CI and meDNA associated with haplotype, but subsequent analysis showed that meDNA did not mediate the relation between haplotype and CI.

**Conclusions:**

Our findings suggest that *CYP17A* associates with PFC-dependent cognitive deficits in schizophrenia but did not support the hypothesis that CA plays a role in this association via meDNA or any other mechanism. Grant support: 21-15-00124/Russian Science Foundation https://rscf.ru/project/21-15-00124/.

**Disclosure:**

No significant relationships.

